# Safety and effectiveness of balloon-expandable Myval transcatheter aortic valve implantation: a single-center, real-world evidence from Kazakhstan

**DOI:** 10.1186/s13019-026-04087-9

**Published:** 2026-04-15

**Authors:** Abdurashid Mussayev, Serik Alimbayev, Zhanar Amanzholova, Nursultan Tanaliyev, Aknur Kali, Muradym Murzagaliyev, Dinmukhamed Seidanov, Almas Zhangirkhanuly Bimakhan, Timur Lesbekov, Tolganay Kamzayeva, Nursultan Orynbassar, Yerkezhan Raissov, Yuriy Pya

**Affiliations:** 1 Department of the Catheterization Laboratory, University Medical Center, Astana, 010000 Kazakhstan; 2 Department of Interventional Cardiology, University Medical Center, Astana, 010000 Kazakhstan; 3 Department of Adult Cardiac Surgery, University Medical Center, Astana, 010000 Kazakhstan; 4 Department of Anesthesiology, University Medical Center, Astana, 010000 Kazakhstan; 5 Department of Radiology and Nuclear Medicine, University Medical Center, Astana, 010000 Kazakhstan

**Keywords:** Aortic stenosis, TAVI, Transcatheter heart valve, Myval, Real-world study, Kazakhstan

## Abstract

**Background:**

Untreated aortic stenosis (AS) leads to significant mortality and morbidity. Balloon-expandable Myval transcatheter heart valve (THV) has demonstrated safety and effectiveness for treating severe AS in patients at intermediate or high risk for surgery. This retrospective observational study aimed to analyze the safety and efficacy of Myval THV in AS patients who underwent transcatheter aortic valve implantation (TAVI) at a single-center.

**Methods:**

Data from 100 consecutive patients who underwent transfemoral TAVI for severe symptomatic AS with Myval THV were analyzed. Baseline characteristics including medical history, clinical features, procedural data, laboratory and echocardiographic data, and outcome data at discharge and 30 days were collected retrospectively. Outcomes as defined according to the consensus document of the Valve Academic Research Consortium-3 were determined.

**Results:**

Baseline characteristics of 100 patients were: 64% males, mean age: 70.87 ± 7.85 years, mean body mass index: 27.97 ± 4.30 Kg/m^2^, Society of Thoracic Surgeons risk score: 2.88 ± 2.18% and log EuroSCORE: 3.34 ± 2.82% respectively. Following transfemoral TAVI, mean and peak gradients were reduced (*p* < 0.001). There was a significant improvement in Vmax 2.50 ± 1.52 m/s (*p* < 0.0001), left ventricular ejection fraction 50.5 ± 10.64% (*p* = 0.0003), aortic valve area 1.76 ± 0.52 (*p* = 0.0013), and indexed aortic valve area 0.92 ± 0.28 (*p* = 0.0034) at discharge which continued at 30-day follow-up. Nearly 94% of patients were asymptomatic and in the New York Heart Association class I–II, with 22% of patients reduced the degree of mitral regurgitation at 30 days. At discharge, only 4.12% had moderate aortic regurgitation while 73.2% had none. Two patients had stroke while 15 patients had conduction disturbances, which led to the implantation of permanent pacemakers. No death or hospitalization were reported. Life-threatening bleeding and access-related complications did not occur in any patient. The rates of the device and technical success were 95%.

**Conclusion:**

Real-world data on the use of technologically advanced Myval THV leads to safe and precise orthotopic positioning in TAVI patients, ensuring optimal, large effective orifice area, and normal hemodynamic status.

## Background

Advent and use of transcatheter heart valve (THV) interventions have improved the outcomes of patients with valvular heart disease. The use of transcatheter valves in patients unfavorable to surgical intervention is a recommended option [1]. Aortic stenosis (AS) if left untreated leads to significant mortality and morbidity [2]. The management of AS has changed significantly with the advent of transcatheter aortic valve implants [3]. The guidelines suggested treatment for severe calcific AS in older people is transcatheter aortic valve implantation [4]. Despite being recommended initially for the treatment of high-risk or inoperable patients, it has also demonstrated superiority in low-risk surgical patients [5]. Recent recommendations state, that transcatheter aortic valve implantation (TAVI) has a lower mortality risk and is associated with a shorter hospital length of stay, more rapid return to normal activities, lower risk of transient or permanent atrial fibrillation (AF), less bleeding, and less pain than surgical aortic valve replacement [6]. With more patients to treat with widening indications, transcatheter aortic valve implantation should be safer for the patient and less complex for the operator.

The Myval THV (Meril Life Sciences Pvt. Ltd., Gujarat, India) is a Conformité Européenne- marked, newer-generation balloon-expandable TAVI system. It has demonstrated safety and effectiveness for the treatment of severe AS in patients at intermediate or high risk for surgery [7]. In the first-in-human study, Myval THV demonstrated the primary safety and efficacy with no new permanent pacemaker requirement for up to 12-month follow-up [8]. This MyVal-1 study was then extended to 100 patients. At 6-month follow-up, 6-minute walk test and Kansas City Cardiomyopathy Questionnaire scores improved when compared with baseline [9]. In a retrospective analysis of 68 patients who underwent Myval THV implantation in bicuspid aortic valve at 12 European centers, at 30 days, only 3% cases had greater than mild aortic regurgitation (AR). There were no procedural complications, no coronary obstruction, no aortic root injury, no device embolization, no procedural death and no stroke. All-cause death was 3% and the permanent pacemaker implantation (PPI) rate was 8.5% [10]. Retrospective non- randomized comparative analysis and registries have demonstrated favorable results and potential advantages of Myval THV over the current generation of THVs [4]. With more than 1,800 implants across the globe and experience from unpublished registries have shown successful results with ease of deployment in a wide range of complex anatomies, including bicuspid aortic valves with low complication rates [11]. To dwell on this evidence, a real-world, retrospective observational safety and efficacy study was conducted at a single center on 100 patients who underwent TAVI with the Myval THV system.

## Methods

### Patient population

This is a retrospective, observational, single-arm and single-center study. Data from 100 consecutive patients who underwent transfemoral TAVI from October 2019 to January 2023 at a single center (UMC Heart Center) were included as the study population. Being a retrospective study, it was exempted from the informed consent process and data was collected anonymously according to the study inclusion criteria in adherence to the recent guidelines’ recommendations [12, 13]. Data from the patients who underwent TAVI for severe symptomatic AS with Myval THV were collected. The study was approved by the Institutional Ethics Committee and was performed according to the guidelines provided by the committee.

Baseline characteristics including medical history, clinical features, procedural, echocardiographic, and outcome data at 30 days were collected retrospectively.

### Echocardiographic assessment

As per the American Society of Echocardiography guidelines, the transthoracic echocardiograms of the study population were conducted at baseline, post-procedure, and 30 days following the index procedure [14]. With the aid of echocardiography, severe AS was defined as an aortic valve area (AVA) of ≤ 1 cm^2^ (or an indexed AVA ≤ 0.6 cm^2^/m^2^) and/or a mean pressure gradient of ≥ 40 mmHg or a peak aortic velocity ≥4 m/s. The left ventricular systolic function and device hemodynamic assessments were assessed and graded using transthoracic echocardiography according to established guidelines [15–17].

### Procedure

The TAVI procedures were performed by the same team in the same catheterization laboratory at a single center. Before the procedure, both the aortic valve and the vascular access were evaluated using multi-slice computed tomographic angiography of the entire aorta with vascular window settings in all the patients. Transfemoral access was approached in all patients. The study subjects underwent TAVI procedure using Myval THV.

### Outcomes

Outcomes as defined according to the consensus document of the Valve Academic Research Consortium-3 (VARC-3), were determined as in-hospital death and 30-day all-cause mortality, neurologic events, hospitalization (or re-hospitalization), bleeding and transfusion, vascular and access-related complications, cardiac structural complications, other procedural or valve-related complications, new conduction disturbances and arrhythmias, myocardial infarction, valve dysfunction, acute kidney injury, new PPI, device success, technical success and early safety [15]. Echocardiographic changes and improvement in New York Heart Association (NYHA) functional improvement at 30 days were also analyzed.

### Device description

Myval THV is a novel balloon-expandable THV system, built on a nickel-cobalt alloy (MP35N) frame offering optimal radial strength and radiopacity. It is crafted as a tri-leaflet honey-comb scaffold from decellularized bovine pericardium tissue. The design preserves coronary flow as well as provides high radial strength at the annular base. The design of the Myval THV ensures its orthotopic deployment. The THVs are available in different sizes: conventional (20, 23, 26, and 29 mm), intermediate (21.5, 24.5, and 27.5 mm) and extra-large (30.5 and 32 mm) and are compatible with 14-Fr introducer sheath. The navigator balloon-expandable THV delivery system ensures trauma-free negotiation across the aortic arch and minimizes or eliminates the risk of a periprocedural stroke during arch navigation.

### Statistical analysis

Data were analyzed using SPSS version 22.0 (SPSS Inc. Armonk, New York, USA). Continuous data are presented as mean ± standard deviation (SD) for parametric variables, and median with interquartile range for nonparametric variables. Categorical variables are presented as frequencies and percentages and were compared using Fisher’s exact, and chi- square tests. Two-tailed p value < 0.05 were considered statistically significant.

## Results

### Baseline characteristics

The baseline demographic, laboratory and clinical characteristics of the patients are shown in Table 1. The mean age of the study population was 70.87 ± 7.85 years, of which 62% were males. The average body mass index was 27.97 ± 4.30 Kg/m^2^. Laboratory investigations were near normal in all patients. The mean Society of Thoracic Surgeons (STS) score of the group was 2.88 ± 2.18% while log EuroSCORE was 3.34 ± 2.82%.

**Table 1:** Demographic, laboratory and clinical parameters

Characteristic	N = 100
Age, years	70.87 ± 7.85
**Gender**	
Female, n (%)	38 (38.00)
Male, n (%)	62 (62.00)
BMI, Kg/m^2^	27.97 ± 4.30
Serum creatinine, mg/dL	1.03 ± 0.3
STS Score	2.88 ± 2.18
log EuroSCORE	3.34 ± 2.82

Data is shown as mean ± SD or n (%)

BMI, body mass index; log EuroSCORE, logistic European System for Cardiac Operative Risk Evaluation; STS, society of thoracic surgeon’s score

The most prevalent medical history (Table 2) included hypertension (77%), cerebrovascular disease (35%) and diabetes (24%). Among the study patients, 3% had a previous coronary artery bypass graft surgery (CABG), 13% had previous percutaneous coronary intervention (PCI), and 4% had stroke/transient ischemic attack. Aortic degenerative surgical valves were performed in 4% of patients. Prior to TAVI, 9% patients had a diagnosis of AF, 3% had atrio-ventricular (AV) block, 5% had left bundle branch block (LBBB), 2% had right bundle branch block (RBBB), and 1% had permanent pacemaker (PM) (Table 2). The Multislice computed tomography findings revealed bicuspid aortic valve morphology in 25% of patients. The mean iliofemoral arterial diameter was 7.7 ± 0.5 mm. The mean right and left coronary artery ostial heights were 16.7 mm and 13.7 mm, respectively.

**Table 2:** Baseline medical history, conduction disturbances, and MSCT findings of study subject

Medical history [n (%)]	N = 100
Diabetes mellitus	24 (24.00)
Hypertension	77 (77.00)
Cerebrovascular disease	35 (35.00)
Peripheral vascular disease	21 (21.00)
Cancer history	11 (11.00)
Previous CABG	3 (3.00)
Previous PCI	13 (13.00)
Previous Stroke/TIA	4 (4.00)
Previous valve surgery	6 (6.00)
**Conductance disturbances**	**n (%)**
Atrial Fibrillation	9 (9.00)
AV block	3 (3.00)
LBBB	5 (5.00)
RBBB	2 (2.00)
PM	1 (1.00)
Pericardial effusion	9 (9.00)
**MSCT findings**	
Bicuspid aortic valve morphology	25 (25.00)
Iliofemoral arterial diameter, mean ± SD	7.7 ± 0.5 mm
Mean LCA height	13.7 mm
Mean RCA height	16.7 mm

Values are given as n (%)

AV Block, atrioventricular block; CABG, coronary artery bypass graft; LBBB, left bundle branch block; LCA, left coronary artery; MSCT, Multislice computed tomography; PCI, percutaneous coronary intervention; RBBB, right bundle branch block; RCA, right coronary artery; TIA, transient ischemic attack

### Procedural details

All patients underwent TAVI procedure via the transfemoral route. Pre-implantation balloon dilatation was performed in 39% of patients. None of the patients required post-dilatation. The 26 mm valve size was deployed in 27% of patients, 19% had 29 mm while 17% had 23 mm valve size, and 28% of patients were implanted intermediate sizes (21.5 mm, 24.5 mm, and 27.5 mm) of balloon-expandable valve. In one patient two devices were used, Portico (27 mm) and Myval THV (24.5 mm) respectively, because of dislocation of the first valve to ascending aorta. Following TAVI, mean and peak gradients were reduced. The procedural details are placed in Table 3.

**Table 3:** Procedural details

Procedural parameters	n (%)
Pre-dilatation	39 (39.00)
Post-dilatation	0 (0.00)
**Myval THV size (mm)**,*N* = 100	
20	2 (2.00)
21.5	4 (4.00)
23	17 (17.00)
24.5	14 (14.00)
26	27 (27.00)
27.5	10 (10.00)
29	19 (19.00)
30.5	2 (2.00)
32	5 (5.00)

### Hemodynamic outcomes at discharge and 30-day follow-up

**Fig. 1:**
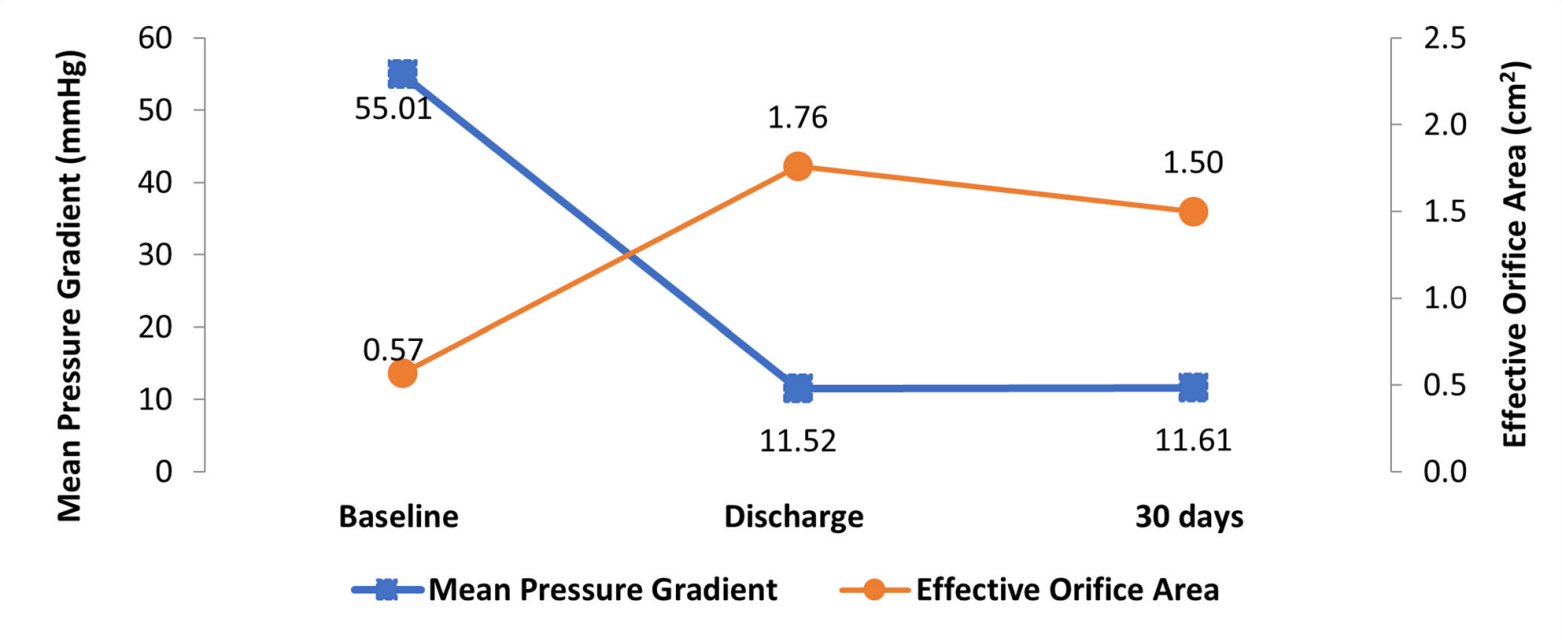
Comparison of hemodynamic outcome

Table 4 describes the comparative echo findings at baseline, discharge and 30-day follow-up. Following TAVI, there was a significant improvement in left ventricular ejection fraction 50.5 ± 10.64% (*p* = 0.0003), AVA 1.76 ± 0.52 (*p* = 0.0013), and indexed AVA 0.92 ± 0.28 (*p* = 0.0034) at discharge which continued at 30-day follow-up. The mean gradient significantly (*p* < 0.001) decreased to 11.61 ± 4.98 mmHg (Fig. 1). When compared to baseline at 30 days, there was significant (*p* < 0.0001) improvement in Vmax (m/s) (4.63 ± 0.84 Vs. 2.50 ± 1.52). Also, none of the patients had severe mitral regurgitation at discharge and 30-day follow-up (Table 4).

**Table 4:** Comparative echocardiographic findings at baseline, discharge and 30 days

Parameters	Baseline	Discharge	30-day follow- up	*P*-value (Baseline vs. 30 days)
LVEF (%)	50.03 ± 12.67	50.54 ± 10.64	56.08 ± 8.97	0.0003*
Aortic valve area (cm2)	0.57 ± 0.17	1.76 ± 0.52	1.50 ± 0.34	0.0013*
Indexed aortic valve area (cm2/m2)	0.32 ± 0.10	0.92 ± 0.28	0.82 ± 0.21	0.0034*
Mean aortic valve gradient (mmHg)	55.01 ± 16.38	11.52 ± 4.12	11.61 ± 4.98	< 0.0001*

Values are given as mean ± SD

Paired Student’s t-test, *P < 0.005, statistically significant difference between baseline and 30-day follow-up

LVEF, left ventricular ejection fraction

**Fig. 2:**
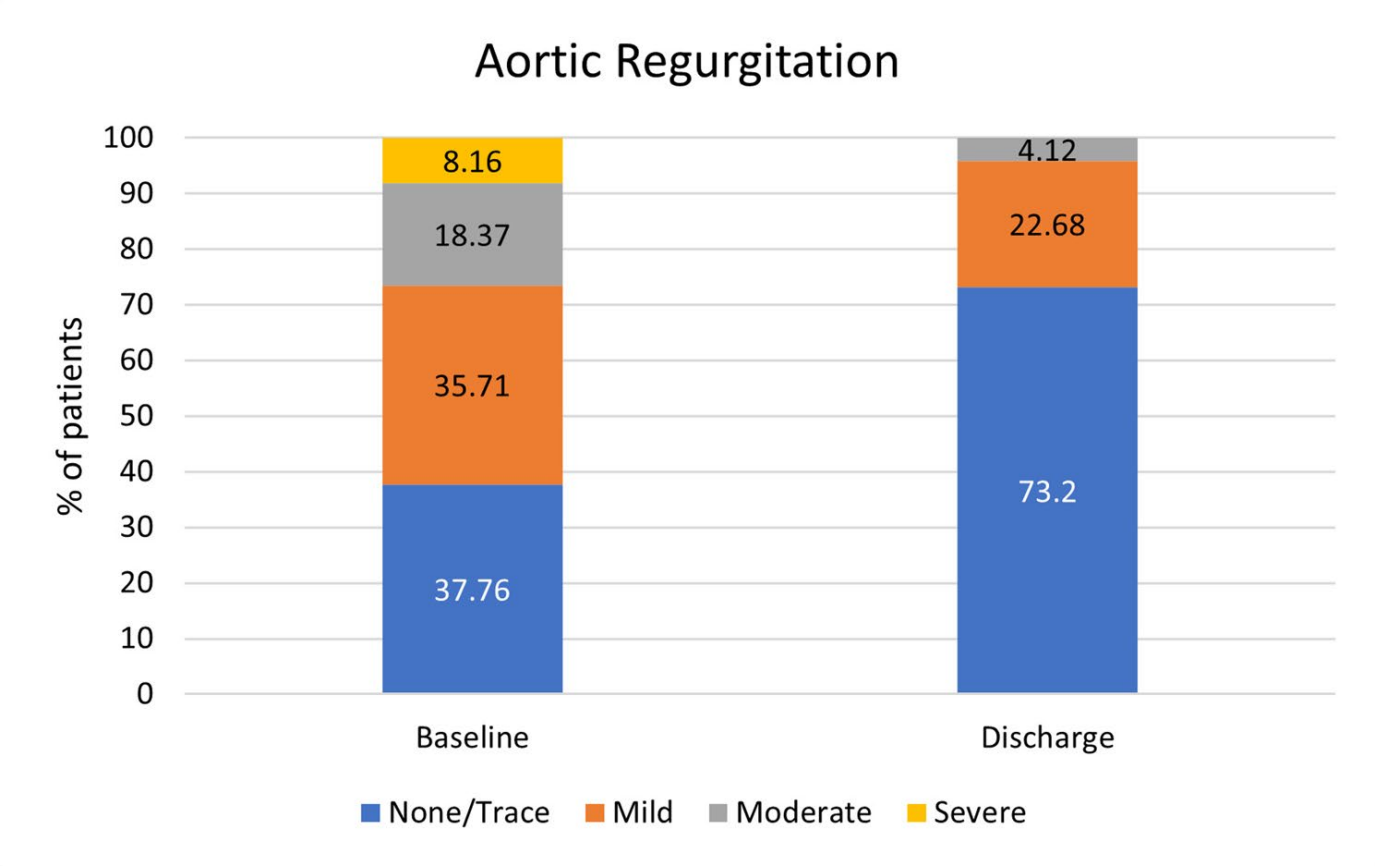
Percentage of patients with aortic regurgitation at baseline and discharge

At baseline, 35.71% of patients had mild, 18.37% had moderate and 8.16% had severe AR. At discharge, there was improvement with only 4.12% having moderate, and 73.2% with no AR (Fig. 2).

**Fig. 3:**
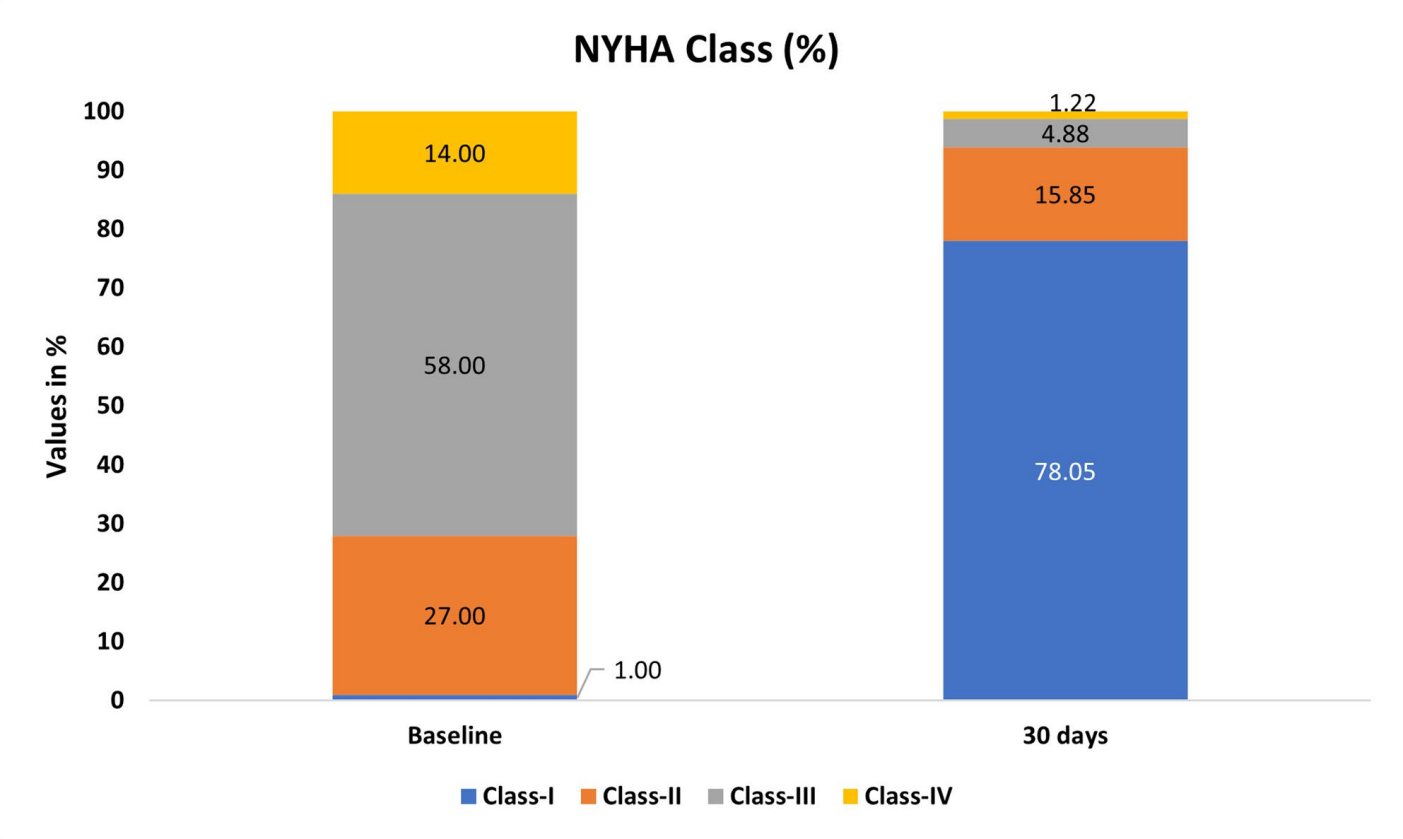
Improvement in New York Heart Association (NYHA) functional class from baseline to 30- day follow-up

According to the NYHA functional class, all patients at baseline were symptomatic, with 72% in class III, and IV. At 30 days, a significant (*p* < 0.005) improvement in NYHA class was noted. Nearly 94% of patients were asymptomatic and in class I–II (Fig. 3).

### Clinical outcomes

Table 5 depicts clinical outcomes at 30 days. No death was observed through 30-day follow-up. Minor vascular complications were observed in 1 patient. Life-threatening bleeding, access-related complications or acute kidney injury did not occur in any patient (Table 5). Two patients had strokes, one of them was a valve-in valve procedure. Total 15% of patients were implanted with a new permanent pacemaker after the procedure. One patient had a myocardial infarction, because of migration of calcium to left main artery, while 4% had conductance disturbances (Table 5). According to VARC-3 criteria, technical success and device success were achieved in 95% of patients, and early safety was achieved in 79% of patients.

**Table 5:** Clinical outcomes at 30-day follow-up

Complication [n (%)]	N = 100
All-cause mortality	0 (0.00)
Minor vascular complications	1 (1.00)
Bleeding	0 (0.00)
Stroke/TIA	2 (2.00)
Acute kidney injury	0 (0.00)
Myocardial Infarction	1 (1.00)
Conduction disturbances	4 (4.00)
New Permanent Pacemaker Implantation	15 (15.00)
Surgery or intervention related to device	4 (4.00)

Values are represented as n (%)

TIA, transient ischemic attack

## Discussion

TAVI has become the standard of care for patients with symptomatic severe AS. Continuous improvements in technology over the earlier generations of THVs have led to improved efficacy and safety, making this technology available to the wider population [2]. The development of new generation THVs like Myval THV makes life-saving procedures for difficult and unusual anatomies easily accessible [4]. Early results from the MyVal-1 first-in-human trial are promising, with excellent procedural success, precise deployment, and good outcomes in short-term follow-up [8]. The results are also in concurrence with other retrospective non-randomized comparative analysis and registries, inferring favorable outcomes and potential advantages of Myval THV [11].

In the present real-world retrospective study, analyses showed the mean age of the patients at this center was 70.87 ± 7.85 years, with a mean STS score of 2.88 ± 2.18%. Surgical/interventional history of these 100 TAVI patients included 6% valve surgery, 3% CABG and 13% PCI. Before TAVI, an electrocardiogram revealed, that 2% had a diagnosis of RBBB, 3% of AV block, 5% of LBBB, and 9% had a diagnosis of AF and pericardial effusion respectively. In 39% of patients, pre-dilation of the native aortic valve was performed based on computed tomographic data. Pre-dilatation is mostly used for easier valve crossing in patients with severe calcifications and very narrow valve orifices. After implantation, none required post-dilatation. This retrospective analysis is well in accordance with the study reported by García-Gómez et al. wherein 100 patients were also studied retrospectively at nine European centers with a mean age of 80 ± 6.5 years and mean STS of 2.4 ± 0.8% [18].

In accordance with the findings of the first-in-human MyVal-1 study, there was a significant (*p* < 0.05) improvement in the mean aortic gradient and AVA [8]. Device and technical success were 95%. As per VARC-3 technical success, there were no cases of valve embolization, annulus rupture, or procedural death. Echocardiographic and functional improvement was maintained at 30 days and there were no deaths. There was significant improvement in the NYHA class, with 94% of patients being asymptomatic at 30 days. Vascular complications were observed in only one patient while 2 patients had stroke. Life-threatening bleeding complications did not occur in any patient. The VARC-3 outcomes of this retrospective data are in line with those reported in the first-in-human MyVal-1 study, possibly attributed to the TAVI procedures performed by the same operators without complexity, in the same catheterization laboratory at a single-center and device evolution [8].

Since Myval THV is available in conventional, intermediate and extra-large sizes, it enables the interventional cardiologists to choose the most appropriate valve size. In a retrospective survey conducted in 27 countries on 1,115 patients who underwent Myval THV implantation, it was demonstrated that intermediate Myval THV sizing was used in 42% of patients. In this contemporary real-world practice, 65% patients had conventional sizes, 30% had intermediate and 7% had extra-large Myval THV sizing, addressing the unmet need of the interventional cardiologists for a range of more calibrated THV sizing [19]. In a comparative retrospective core lab analysis of quantitative video-densitometric aortography, Myval THV had the least moderate/severe quantitative AR (2.8%) compared to Sapien 3 THV (8.3%) and Sapien XT THV (10.9%) [20]. None of the patients in the present analysis had severe mitral regurgitation at 30 days and at discharge, 73.2% had no AR. This could be due to its external skirt design minimizing paravalvular leak as well as the availability of intermediate and extra-large sizes, which helps achieve a more accurate and optimal fit for the native annulus geometrics.

In a comparative blinded echocardiographic analysis of Myval and Sapien valves on a matched population of 103 patients, at 30 days there was a lower need for PPI (5.8% versus 15.5%, *p* = 0.02) and significantly lower mean gradients and paravalvular leaks in the Myval group compared to the Sapien 3 group [21]. In the European registry of 1,131 TAVI patients reported 7.4% of pacemaker implantation with Myval THV [22]. Similarly, a retrospective study involving an overall population of 180 patients reported 15.4% of PPI among 110 patients treated with the Myval THV [23]. Similar findings were published in the EVAL registry performed on a retrospective analysis of 166 consecutive patients undergoing TAVI with either Myval THV (*n* = 58) or Evolut R THV (*n* = 108), wherein Myval group had a significantly lower incidence of moderate paravalvular leak (6.9% versus 19.8%, *p* = 0.039) and PPI (11% versus 27.5%, *p* = 0.02) [24]. In the present study, PPI rate was 15% at 30 days, which is consistent with these previously reported findings.

Results of two ongoing prospective, large, randomized studies: the LANDMARK trial and the COMPARE-TAVI trial are evaluating its performance and long-term durability against commonly used self-expandable and balloon-expandable THV which will further infer its potential [4, 25]. In the LANDMARK trail a total of 768 patients (mean age 80 years) having transfemoral TAVI at low surgical risk were randomly assigned 1:1 to receive a Myval THV implant or a different modern TAVI device (Sapien (BEV) and Evolut (SEV)). In comparison to other modern THVs, the Myval THV was found to be non-inferior at 30 days, as evidenced by the primary composite endpoint occurring in 24.7% and 27.6% of the Myval and control groups, respectively (risk difference − 2.7%; one-sided upper 95% CI 3.6%; *p* < 0·0001 for non-inferiority). No one major endpoint component showed any discernible variations, and there were no variations in terms of technical or device success (as determined by the VARC-3 criterion). Additionally comparable were secondary objectives such as pacemaker insertion rates and improvements in hemodynamic characteristics (effective orifice area and mean pressure gradient).

While these short-term results are encouraging, more information about hemodynamic performance and the likelihood of structural valve deterioration over a longer period of time will be necessary to verify the Myval THV’s durability when compared to more modern valves.

This study has several limitations. It is a retrospective, single-center, single-arm analysis with a relatively small sample size and a short follow-up limited to 30 days, which restricts the ability to draw robust conclusions regarding long-term safety, durability, and comparative effectiveness. Additionally, the absence of a comparator group precludes direct comparisons with other contemporary THVs. Therefore, the findings should be interpreted within the context of real-world clinical practice from a Central Asian center in Kazakhstan.

## Conclusion

The technological advancement of Myval THV over the previous generation of THVs leads to simpler and safer procedures. The innovative THV system demonstrates favorable outcomes and potential advantages of Myval THV.

## Data Availability

The datasets used and/or analysed during the current study are available from the corresponding author on reasonable request.

## Abbreviations

THV: Transcatheter heart valve
TAVI: Transcatheter aortic valve implantation
AS: Aortic stenosis
AF: Atrial fibrillation
AR: Aortic regurgitation
AVA: Aortic valve area
CABG: Coronary artery bypass graft surgery
PCI: Percutaneous coronary intervention
LBBB: Left bundle branch block
RBBB: Right bundle branch block
NYHA: New York Heart Association
STS: Society of Thoracic Surgeons
TAVR: Transcatheter aortic valve replacement
VARC-3: Valve Academic Research Consortium-3
